# Evidence for the intermediate disturbance hypothesis and exponential decay in replacement in *Streptococcus pneumoniae* following use of conjugate vaccines

**DOI:** 10.1038/s41598-022-11279-5

**Published:** 2022-05-07

**Authors:** A. Cristina Paulo, Raquel Sá-Leão

**Affiliations:** Laboratory of Molecular Microbiology of Human Pathogens, Instituto de Tecnologia Química e Biológica António Xavier, Universidade Nova de Lisboa, Oeiras, Portugal

**Keywords:** Bacteria, Infectious-disease epidemiology, Microbiology, Microbial ecology

## Abstract

Understanding how pneumococci respond to pneumococcal conjugate vaccines (PCVs) is crucial to predict the impact of upcoming higher-valency vaccines. However, stages in pneumococcal community succession following disturbance are poorly understood as long-time series on carriage are scarce and mostly evaluated at end-point measurements. We used a 20-year cross-sectional dataset of pneumococci carried by Portuguese children, and methods from community ecology, to study community assembly and diversity following use of PCV7 and PCV13. Two successional stages were detected upon introduction of each PCV: one in which non-vaccine serotypes increased in abundance, fitted by a broken-stick model, and a second in which the community returned to the original structure, fitted by a geometric series, but with different serotype profile and a drop in richness as great as 24%. A peak in diversity was observed for levels of intermediate vaccine uptake (30–40%) in agreement with the intermediate disturbance hypothesis. Serotype replacement was fitted by an exponential decay model (*R*^2^ = 80%, P < 0.001). The half-life for replacement was 8 years for PCV7 and 10 years for PCV13. The structure of the pneumococcal community is resilient to vaccine pressure. The increasing loss of diversity, however, suggests it could eventually reach a threshold beyond which it may no longer recover.

## Introduction

*Streptococcus pneumoniae* (pneumococcus) is a common member of the upper respiratory tract microbiota of humans and is mostly found in the nasopharynx of children^1^. Pneumococcus carriage is predominantly asymptomatic and occurs multiple times throughout life in virtually all individuals^2–4^. Its prevalence, however, is higher in young children: it has been estimated that approximately 50–60% of the worldwide pediatric population is colonized with pneumococci^5,6^. Several studies indicate that children are the main reservoirs and spreaders of pneumococci in the community^3,7^.

Pneumococci can cause a diverse spectrum of diseases that range from otitis media and pneumonia to invasive pneumococcal disease (IPD) such as meningitis, empyema, sepsis and bacteremic pneumonia, that, together, are associated with significant mortality and morbidity: in 2015, for example, a conservative total of 318,000 deaths and 3.7 million cases of severe pneumococcal disease were estimated to have occurred in children aged 1–59 months worldwide irrespective of their HIV-status^8^.

In 2000, the first pneumococcal conjugate vaccine (targeting seven serotypes, PCV7) was licensed and introduced in the national immunization programs (NIP) of the USA and, subsequently, in several other countries^9,10^. Within 3 years of PCV7 introduction, a significant reduction of IPD incidence was observed, ranging between 38 and 80%, depending on the country^11^.

Introduction of PCV7 led to an increase in the circulation of serotypes not targeted by PCV7. In 2010, an expanded version of PCV7, targeting PCV7 serotypes plus 6 additional serotypes (PCV13), was licensed simultaneously in the USA and in the European Union^12^. PCV13 improved significantly the potential coverage of serotypes causing IPD and being commonly carried^8,12^. With both vaccines, extensive serotype replacement with serotypes not targeted by the vaccine was observed in colonization; partial replacement, with varying magnitudes across countries, was observed in non-invasive disease and in IPD^8,13–18^.

A third generation of extended-valency pneumococcal conjugate vaccines should become available soon^19^. Whilst serotype replacement seems to be a general response to PCVs, the magnitude of expansion of non-vaccine serotypes is unknown as well as the time elapsing until the pneumococcal population recovers from PCVs disturbance. In ecology, disturbances are often used as an experimental tool to understand how a community works^20^. The use of PCVs on the pneumococcal community is thus a broadly ecological quasi-experiment.

In Portugal, where pediatric pneumococcal carriage has been extensively studied since 1996, the prevalence of pneumococcal colonization among children up to 6 years old is around 60%^15,16^. PCV7 was available in Portugal since 2001 and was replaced by PCV13 in 2010. Until 2015, PCVs were available in the private sector, its full costs being supported by the children’s guardians. The proportion of children vaccinated with PCVs increased over time. By 2003, it was estimated that 56% of the < 1 year old cohort was vaccinated with PCV7 increasing to 79% by 2007^15^. Unsurprisingly, a significant impact in vaccine-type carriage and disease was observed^15,16,18^. In July 2015, PCV13 was introduced in the NIP^21^.

In this paper we used tools from community ecology—measures of diversity, and variation in serotype abundance, in serotype composition and community assemblage—to understand how the pneumococcal population structure is shaped following introduction of PCVs. For that, we used a 20-year (1996–2016) time series of pneumococcal carriage prevalence studies conducted in Portugal among children up to 6 years old since the pre-vaccine era^15,16^.

## Results

### Study characterization

The population under study was described in detail elsewhere^15,16^. Supplementary Table 1 summarizes the most relevant characteristics. From 1996 to 2016, 8,472 nasopharyngeal samples were collected from children attending 56 day-care centers. Overall, the children’s mean age was 3.4 ± 1.5 years old and did not vary between calendar years, except for 2012 (3.0 ± 1.5), 2015 (2.9 ± 1.4) and 2016 (2.8 ± 1.6). In these years, on average, the proportion of children older than three years old that were enrolled was significantly lower (P = 1.87e−04, P = 1.07e−07 and P = 1.62e−08, respectively, Welch two-sample t-test FDR adjusted). These differences are, nonetheless, in the range of a few months and thus with no biological impact for pneumococcal carriage. The ratio of males to females was around 1, and no significant differences were found between calendar years (P = 0.221, Pearson's chi-squared test).

Overall, pneumococcal carriage prevalence was 61.3% [95% CI 60.3–62.3]. Compared to the global average, in 2002 (66.7%, [63.1–70.0]), in 2003 (72.7%, [69.1–76.1]), and in 2006 (69.9%, [65.8–73.7]), the prevalence of carriage was significantly higher (P = 1.29e−02, P = 7.94e−08, and P = 3.93e−04, respectively, Pearson's chi-squared test FDR adjusted). There was no correlation between prevalence of carriage and sample size (P = 0.741, Pearson's product-moment correlation).

PCVs uptake increased significantly from 11.6% [95% CI 9.4–14.1] in 2002 to 78.3% [74.8–81.4] in 2009 (P < 2.20e−16, Chi-squared test for trend in proportions). From 2010 to 2012, PCVs uptake remained constant with a mean value of 75.9% [71.3–79.9] (P = 0.975), increasing significantly in 2015–16 to a mean value of 79.6% [74.6—83.7] (P = 0.018). PCV13 was introduced in 2010. In 2011 and in 2012, the proportion of children receiving at least one dose of PCV13 was 24.3% [20.1–29.2] and 55.9% [49.0–62.5], respectively. In 2015 and in 2016 all children received at least one dose of PCV13.

### Pneumococcal serotype diversity

To investigate changes in pneumococcal serotype diversity over time, estimates of Hills numbers ^*0*^*D*, ^*1*^*D*, and ^*2*^*D* by year were obtained using 1,000 bootstrap re-samplings of 275 children (that could be colonized or not with pneumococci). The sample size of 275 was chosen given that it corresponded to the minimum yearly sample size, which occurred in 2012 (Supplementary Table S1). Yearly rarefaction curves were estimated to inspect if the serotypes richness reached an asymptote at this sample size (Supplementary Fig. S1).

**Figure 1:**
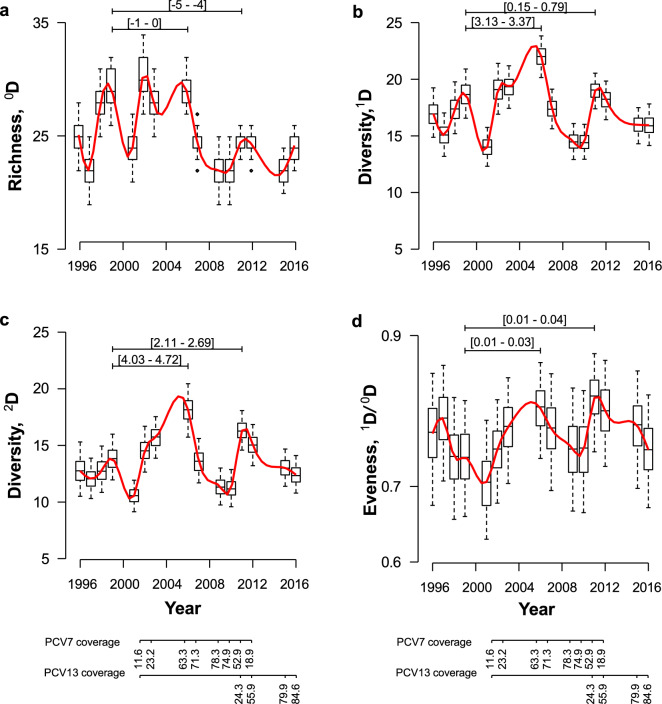
Estimates of pneumococcal serotype diversity per sampling year. (**a**) Richness, ^*0*^*D*; (**b**) Diversity, ^*1*^*D* (exponential of Shannon’s diversity); (**c**) Diversity, ^*2*^*D* (inverse of Simpson’s index); (**d**) Evenness, ^*1*^*D/*^*0*^*D*. For each year a boxplot summarizes the data corresponding to estimates of diversity derived from 1,000 bootstrap re-samplings of 275 children. A smooth line, in red, was imposed to each plot. The differences between the diversity medians are shown as 95% bootstrap confidence intervals. Pneumococcal vaccine uptake is shown at the bottom of the image.

Richness, ^*0*^*D*, oscillated over time peaking in 1999 (median of 29 serotypes, 95% percentile bootstrap [CI 26–32]), 2002 (30, [27–34]), 2006 (29, [27–32]), and 2011–2012 (25, [23–26]) (Fig. 1a). Compared with the peak in 1999, before PCV7 implementation, a significantly lower median number of serotypes was observed in 2011–12 (4–5 fewer serotypes). The number of serotypes estimated in 2016 was similar to the one estimated for 2011–12. After 2006, the median number of serotypes decreased and reached a global minimum in 2015 with an estimated median of 22 serotypes (95% percentile bootstrap [CI 20–24]), corresponding to a 24.4% drop in richness compared to the pre-PCV7 peak of 1999 (Fig. 1a).

Both Hills numbers ^*1*^*D* and ^*2*^*D* (which correspond to the exponential of the Shannon index, and the inverse of the Simpson index, respectively) showed a peak around 2006 with 86.5 [95% percentile bootstrap CI 75.7–96.8] and 18.2 [16.2–19.9] effective number of serotypes, respectively; and another peak around 2011 with 70.0 [61.9–78.1] and 16.2 [15.0–17.6] effective number of serotypes, respectively (Fig. 1b,c); notably, these peaks were coincident with peaks in ^*0*^*D*. For both ^*1*^*D* and ^*2*^*D* the median values were higher than the ones observed in the pre-PCV7 peak of 1999 (68.0 [56.9–81.6] and 13.8 [12.1–15.5], respectively). In other words, in 2006 the pneumococcal community was about 1.3 (86.5/68.0) and 1.4 (18.2/13.8) fold times more diverse than in 1999. A peak in evenness, ^*1*^*D/ *^*0*^*D*, was observed in 2006 (3.0, [95% percentile bootstrap CI 2.5–3.3]) and in 2011 (2.8 [2.5–3.2]) (Fig. 1d).

**Figure 2:**
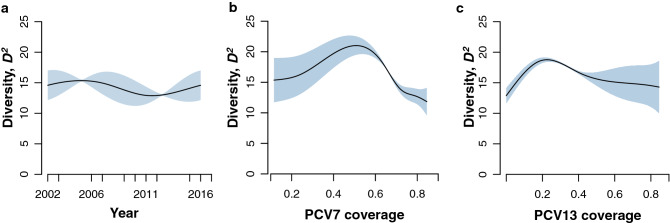
Impact of vaccination on pneumococcal serotype diversity. Partial plots resulting from the fit of a GAM model with three smooth terms to ^*2*^*D* are shown. (**a**) Changes in ^*2*^*D* according to year; (**b**) Changes ^*2*^*D* according to smooth term PCV7 uptake; (**c**) Changes in ^*2*^*D* according to smooth term PCV13 uptake. Smooth functions represent an assembly of polynomials joined together by knots. Each smooth term was significantly associated with ^*2*^*D* (P < 2e-16 for each term).

To interpret the impact of PCV7 and PCV13 on pneumococcal serotype diversity, a GAM model, with smooth functions for year, PCV7 and PCV13 uptake rates, was fitted to the ^*2*^*D* values (Fig. 2a–c). The ^*2*^*D* values were modelled as a Gaussian distribution. After inspecting concurvity, no dependence between the explanatory variables was found (concurvity < 0.5 for all). The model explained about 85.2% of the variance in the data. Each smooth term—year, PCV7 and PCV13—was significantly associated with ^*2*^*D* (P < 2e-16). Inspection of the partial dependent plots showed that PCV7 uptake of around 0.4 led to a peak in ^*2*^*D* (Fig. 2b), whereas for PCV13 the same happened at an uptake of around 0.3 (Fig. 2c).

### Pneumococcal serotype succession and community structure

**Figure 3:**
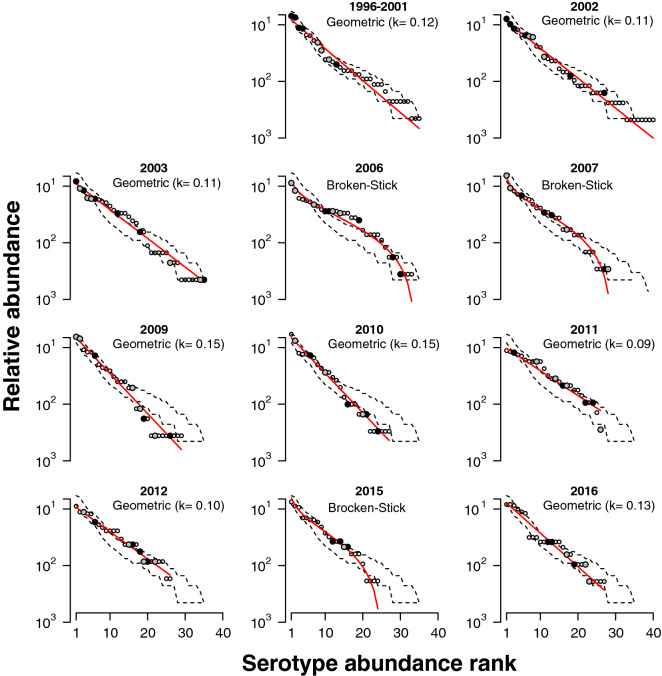
Pneumococcal serotype succession and community structure. Rank abundance curves depicting relative serotype abundance distribution per year. Each panel shows the serotypes (circles) ranked by the logarithm of their relative abundance. Large black circles indicate serotypes targeted by PCV7, large gray circles indicate the six serotypes targeted by PCV13 only, smaller white circles indicate serotypes not targeted by PCV13. Dashed lines in all panels correspond to 95% confidence interval of serotypes abundance during the pre-PCV period (from 1996 to 2001 as indicated in the first panel). Red lines shows the distribution that better fits the data in each year. Below each year, the name of the distribution that better fits the data is indicated. For the geometric distributions, the slope (k) is also indicated.

To investigate the pattern of pneumococcal serotype succession and how it was affected by PCV7 and PCV13 uptake, rank-abundance curves (RAC) were obtained. The serotypes-abundance distributions (SAD) that better fitted the RAC were the geometric series and the Broken-stick models (Fig. 3). After pooling the pre-PCV years, we found the best fit to the pre-PCV7 RAC to be a geometric series distribution with a slope of k = 0.12. As PCV7 uptake increased, a shift from the geometric series to the broken-stick model was observed in 2006–2007. From 2007 to 2010, the pneumococcal serotype community recovered back to a geometric series albeit with a higher slope (k = 0.15) than the one observed in the pre-PCV era. The increase in the slope could be attributed to rare serotypes that became even rarer (below the pre-PCV7 95% CI) and on the increased proportion of dominant serotypes (above the pre-PCV7 95% CI). After PCV13 introduction the pneumococcal serotype community structure initially maintained a geometric series distribution. In 2015, however, it was best described by a broken-stick model. In 2016, it returned to a geometric series described by a slope of k = 0.13, almost identical to the pre-PCV7 slope (k = 0.12) and lower than the pre-PCV13 (k = 0.15). Notably, this was accompanied by a significantly lower number of serotypes as showed in Fig. 1a.

### Similarity decay of pneumococcal serotype communities

**Figure 4:**
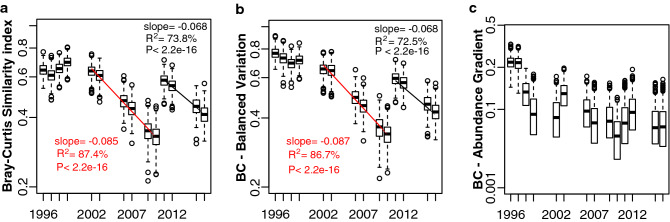
Similarity between pneumococcal communities following introduction of PCVs. (**a**) Bray–Curtis similarity index over time; (**b**) proportion of the Bray–Curtis similarity index explained by serotype replacement (balanced variation in abundance); (**c**) proportion of the Bray–Curtis similarity explained by serotype expassion/reduction (gradient component). For the period 2002–2010, when PCV7 was used, the similarity of each year was compared with the pneumococcal serotype community of 2001; for the period 2011–2016, when PCV13 was used, the similarity of each year was compared with the pneumococcal serotype community in 2010. Boxplots represent the summary of 1000 similarity estimates based on bootstrap re-sampling. In (**a**) and (**b**), for each period an exponential decay model was fitted to the data: the red solid line corresponds to the PCV7 period, the black solid line corresponds to the PCV13 period. The slope, the significance of the model, and the variance (*R*^2^) explained by each model, are indicated.

To compare pneumococcal serotype communities between calendar years, the abundance-based Bray–Curtis similarity index (BC) was used. Pneumococcal community similarity decreased over time (Fig. 4a). To disentangle the relative contribution of serotype replacement and serotype expansion/decrease, for the observed community similarity decay, the corresponding components of the BC, i.e., “balanced variation in abundance” and “abundance gradient”, respectively, were calculated.

Before PCV7 introduction, the “balanced variation in abundance” component of pneumococcal serotype communities (compared to the 2001 community) ranged between 0.61 in 1997 and 0.70 in 1999 (Fig. 4b). After PCV7 introduction, it decreased significantly: the decay in “balanced variation in abundance” between communities of pneumococcal serotypes, from 2002 to 2010, compared with 2001, was characterized by an exponential decay function with a decay rate of − 0.087 that explained about 86.7% of the variability in the data. After PCV13 introduction, serotype replacement occurred again albeit with a significantly lower slope of − 0.068 (P = 1.03e−04, ANOVA), suggesting a slower rate of replacement between 2011 and 2016. Indeed, the replacement half-decay time following introduction of PCV7 in 2001 was estimated as 8.2 years, whereas the replacement half-decay time following introduction of PCV13 in 2010 was estimated as 10.2 years. The contribution of the “abundance gradient” (reflecting expansion or decrease of existing serotypes in the communities) to the BC was less obvious **(**Fig. 4c**)**.

### Composition of pneumococcal serotype communities

**Figure 5:**
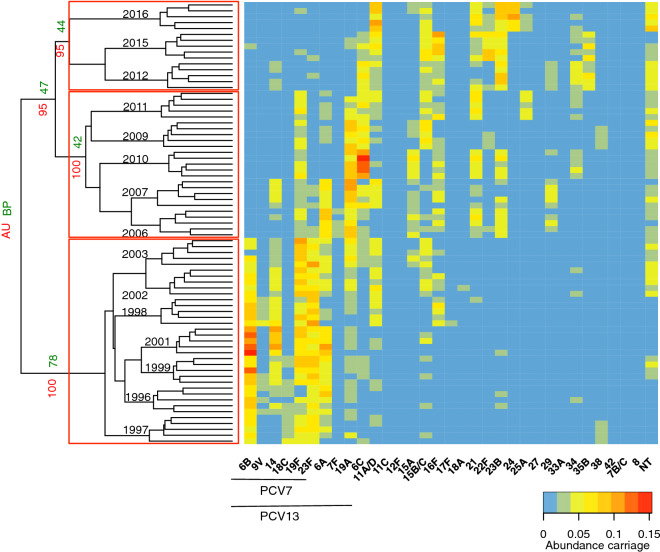
Profiles of pneumococcal serotype communities. Dendrogram (left) depicting the hierarchical clustering of the Bray–Curtis similarity indexes of pneumococcal serotype communities in each year. Three clusters were identified and are highlighted in red. Heatmap (right) shows the relative abundance of each serotype. Green numbers close to dendrogram branches indicate bootstrap probability (BP); red numbers close to dendrogram indicate approximately unbiased P (AU); black numbers indicate study years.

To investigate changes in profiles of serotype community composition, hierarchical clustering of the BC similarity indexes was done. The matrix obtained identified three profiles of pneumococcal serotypes assembly (Fig. 5). The first profile corresponded to the pre-PCV7 period and the initial years in which there was a low PCV7 uptake (2002 and 2003 with 11.6% and 23.2% uptake, respectively). This profile was characterized by a high prevalence of PCV7 serotypes. The second profile corresponded to the calendar years in which PCV7 was established and included the period of 2006 to 2011. This profile was characterized by a substantial decrease of the PCV7 serotypes, a high prevalence of certain PCV13 serotypes, 19A and 7F, and also an increase of certain non-PCV13 serotypes, 15A and 6C (Fig. 5 and Supplementary Fig. S2). The third profile corresponded to the period of 2012 to 2016, which overlapped the PCV13 period when the uptake ranged between 55.9% and 84.6%. This profile was characterized by an overall decrease of the PCV13 serotypes, and an increase of serotypes 15A and 6C as well as other non-PCV13 serotypes, 8, 11A/D, 15B/C, 16F, 22F, 23B, 24, 25A, 34, 35B, and NT (Supplementary Fig. S2).

## Discussion

We used tools from community ecology to analyze a 20-year cross-sectional dataset of pneumococcal carriage by young children living in Portugal and to study how pneumococcal communities respond to disturbances promoted by use of PCV7 and PCV13.

We observed that introduction of PCV7 and PCV13 disrupted the pneumococcal community structure in several ways affecting its diversity, succession patterns, similarity, and composition.

In the pre-vaccine era, the pneumococcal community structure was highly uneven (fitted by a geometric series), which characterized this ecosystem at equilibrium. Introduction of PCV7 triggered a succession-like pattern: the community structure changed giving rise to a community with increased diversity (measured by ^*2*^*D* and ^*1*^*D*) and evenness (measured by ^*1*^*D/*^*0*^*D*) composed of co-dominant pneumococcal serotypes (fitted by a broken-stick model). Notably, at intermediate levels of vaccine uptake, a peak in diversity (^2^*D*) was observed. As years went by, the community structure recovered to a novel equilibrium resembling the pre-PCV pattern (again characterized by a geometric series), albeit with a significant decrease in richness (^*0*^*D,* corresponding to the number of serotypes in circulation). Introduction of PCV13 recapitulated the changes observed upon introduction of PCV7.

In this succession-like pattern, the peak in diversity accompanied by higher evenness (reflected in the hump-shaped ^*2*^*D* curve) at intermediate vaccine uptake, i.e., in 2006 (for PCV7) and in 2011 (for PCV13), are of particular interest as they are in line with the intermediate disturbance hypothesis (IDH)^22–24^.

The IDH was first proposed by Connell^23^ to explain the higher diversity observed on corals from tropical reefs of the outer slopes exposed to storms, as opposed to the lower diversity of the communities of corals living in the inner protected slopes. According to the IDH, diversity is highest at intermediate levels of disturbance due to co-existence of otherwise out-competing species and/or of quicker colonizers; and is low at either extreme due to competitive exclusion or local extinction^22,23^. Although the original theory predicts an increase in richness, other measures, such as evenness, were shown to respond similarly to varying degrees of disturbance. For example, an increase in evenness was shown to occur among vascular plants of the Artic-Alpine tundra due to soil frost disturbance^25^. Such increase was explained by a decrease of highly dominant species that allowed less prevalent species to increase their soil cover. A similar observation was made for the pneumococcal community: at intermediate levels of vaccine uptake of PCV7, a peak in diversity and a higher evenness were observed due to a decrease in the previously dominant PCV7-serotypes and the expansion of non-PCV7 serotypes 19A and 6C^15^. Similarly, an increase in non-PCV13 serotypes, namely 11A/D, 15A and 22F, was observed as the previously dominant PCV13 serotype 19A declined^16^. In both cases, co-dominance of vaccine and non-vaccine serotypes was apparent. This was best explained taking into account that PCVs decrease the competitive advantage of vaccine serotypes, allowing an increase in the abundance of less competitive non-vaccine serotypes^26,27^. A peak in diversity was also observed in Massachusetts four years after PCV7 introduction^26,28^. However, no differences were observed immediately after PCV13 introduction^29^. The latter could be explained by the high levels of PCV13 uptake in place.

At higher vaccine uptake, we observed a decrease in richness due to a decrease in the number of serotypes in circulation (PCV serotypes and rarely seen serotypes). This could have potentially result in a lower prevalence of pneumococcal carriage that, in turn, could facilitate invasion of the niche by other species^30^. Although in our setting this was not observed, a decrease in pneumococcal carriage prevalence was observed in Norway and has been attributed to the continued impact of PCVs^30,31^. In addition, increases in *Streptococcus, Haemophilus*, *Staphylococcus,* and *Moraxella* species have been documented^30,32,33^. Whether these changes are only temporary will depend on the intensity of the disturbance promoted by PCVs^20^.

Another interesting result was the observation that the main species abundance distributions, explaining the mechanism by which pneumococcal serotypes partition community resources (i.e., children to be colonized), seem to alternate between the geometric series and the broken-stick model^34^.

The geometric series, which we observed at equilibrium, can be interpreted as corresponding to a deterministic process of resource partition in which the dominant, most competitive species (in this context, the most competitive serotypes) uses a proportion k of the whole initially available resources (children to be colonized), leaving a fraction (1 − k) of children not colonized. The second most dominant species uses the same k fraction of the remaining resources, the third most dominant the same k fraction of what was left by the first two, until all available resources have been distributed. Moreover, the ecological theory behind the geometric series distribution assumes that the first species is limited by a single abiotic resource, whereas subsequent species compete with each other for similar resources^35,36^. This model fits low diversity communities whose assemblages often show strong dominance like communities at early successional stages and those in harsh or isolated environments^34,36^. For example, this species abundance-distribution is characteristic of many terrestrial plant communities during early successional stages, of the bacterial distribution in an epilithic biofilm^37^, and of gut bacterial communities that are under pressure of digestive fluids^38^. Similarly, the nasopharynx is a nutritionally limited environment where the pneumococcal population, when at equilibrium, has a characteristic low diversity dominated by only a few serotypes^39,40^.

The disturbance caused by introduction of PCVs was best described by the broken-stick model. This model is based on the biological reasoning that resources are randomly distributed among species (or serotypes) and in continuous and non-overlapping niches^36^. Within this model we can envisage an empty larger niche, a larger proportion of children that do not carry PCV serotypes due to PCV vaccination, who can become colonized with non-PCV serotypes. These serotypes will initially fragment and occupy the niche at random before competition takes its toll. This may have important consequences since the first serotypes arriving and colonizing vaccinated children may not be the ones that will prevail later on. Indeed, as time went on, we observed that the species-abundance distribution shifted back to a geometric series (in 2009–2010).

The higher slope of the new geometric series was mirrored by a decrease on the number of dominant serotypes and a concomitant increase in their prevalence. To our best knowledge, this phenomenon has not been explicitly reported before in studies on the impact of PCV on the pneumococcal community. Nonetheless, in Massachussets^28,41^, before PCV7, in 1998–1999, the serotype that ranked as first was carried by approximately 12% of the children, whereas seven years later, in 2007, the most abundant serotype was carried by approximately 18% of the children. This result is almost identical to ours, although we cannot ascertain if the serotype distribution could be fitted by a geometric series.

Whether the higher slope observed in the late PCV7-period, characterized by fewer co-dominant serotypes, corresponded to a transient stage in the succession is unknown as introduction of PCV13 occurred in the meantime. Although use of PCV13 was low (~ 25%) in 2011, we cannot rule out its effect on the community succession.

We found that serotype replacement, measured by the Bray–Curtis similarity index, was still going on nine years after PCV7 introduction and six years after PCV13 introduction. This was supported by three observations: (i) richness reached minimums in 2009–2010 and in 2015–2016; (ii) both geometric series that fitted the RACs in 2010 and in 2016 had higher slopes than the pre-PCV distribution; and (iii) serotype replacement did not reach a stable plateau in 2015–2016 as would be expected if no more changes were occurring. We estimated that, in our setting, the median replacement time, which was fairly well fitted by an exponential time-decay model, was of roughly 8 years if only PCV7 was considered and of 10 years if PCV13 was considered. Of note, Klugman and Rodgers^19^ observed that for IPD replacement in the PCV13 era has been slower than in the PCV7 era. Together these observations suggest that, although the exact time span for replacement may not be generalized, a delay in replacement may be expected as new extended-valency conjugate vaccines are introduced. This delay may be a consequence of different rates of dissemination^42^ and/or a decrease in the effective colonization capacity of the remaining, less competitive, non-vaccine serotypes due, for instance, to the expansion of other bacterial species such as other *Streptococcus* species^32^.

Interestingly, in terms of similarity, we observed that two consecutive calendar years were not necessarily characterized by the most similar pneumococcal serotype profiles between them. This observation may reflect the fact that carriage of serotypes exhibits periodic cycles^43^ and thus similar profiles may be cyclic. Nonetheless, this was mainly observed during the pre-PCV7 period. With the disturbance promoted by PCVs, we observed that calendar years sampled close together in time had a more similar community in composition than communities sampled further apart. Moreover, clustering could be explained taking into account the impact of PCV7 and PCV13. A first profile included the calendar years corresponding to a pre-PCV7 period along with a period in which PCV7 uptake was still low (23.2%); a second profile included the calendar years in which the effects of PCV7 produced a shift in the composition of serotypes circulating in the community, and also the first year of use of PCV13 (also with a low uptake of 24.3%); and finally, a third profile in which PCV13 uptake was already significant and increased from 55.9% to 84.6%. Although we could not establish a precise cut-off value, we observed that at a PCV uptake > 50% a shift in serotype composition, abundance and diversity occurred.

It is known from ecology that all communities have a degree of resistance and resilience to disturbances^20^. As the magnitude and intensity of disturbances increases, both resistance and resilience processes can fail^20^. Whether this will happen with the pneumococcal community, following introduction of expanded valency vaccines, remains to be seen. However, the increasing loss of diversity documented here, suggests the species may eventually reach a threshold beyond which it may no longer recover.

This study has some limitations as it is based on results obtained for a specific population originating from a single region. Additional studies will inform whether the observations documented here can be extended to other settings.

In summary, the overall pattern of the pneumococcal community succession after PCV's introduction seems to go through two major stages disrupting the pre-PCV geometric model: (i) an initial stage with a higher evenness and diversity which is characterized by a broken-stick model; (ii) and a later stage where a shift back to the geometric series occurs albeit with a higher slope than previously observed due to a lower number of serotypes in circulation.

This dynamic model of succession is useful to understand pneumococcal community assembly under disturbance by PCVs that are anticipated to soon occur as novel extended-valency conjugate pneumococcal vaccines are licensed.

## Methods

### Study design and sampling

Data on carriage of pneumococcal serotypes originated from repeated cross-sectional surveys that were conducted between January and March from 1996 to 2016^15,16^. The study population consisted of children up to six years old who were attendees at day care centers in the Lisbon region. Nasopharyngeal swabs were obtained in the winter months of January to March. In each year, one nasopharyngeal swab was obtained from each participant as well as demographic (e.g. age and gender) and clinical data (e.g. vaccine status, antibiotic uptake). The study design, the sampling process and a detailed description of the dataset were described elsewhere^15,16^.

Isolation of pneumococci and serotyping followed standard methods as described previously^15,16^. Briefly, nasopharyngeal samples were plated onto blood agar supplemented with 5 µg/mL of gentamicin (GBA) and were incubated overnight in anaerobic jars at 37 °C. Pneumococcal identification was based on colony morphology, occurrence of α-hemolysis and optochin susceptibility. Bile solubility test was performed for optochin resistant isolates. Serotypes were determined by multiplex PCR using primers previously described (primer sequences available at http://www.cdc.gov/streplab/pcr.html). When negative or inconclusive PCR results were obtained, the Quellung reaction was performed using specific antisera (Statens Serum Institute, Copenhagen, Denmark)^44^. Unencapsulated strains were identified using a multiplex PCR-based strategy as previously described^45^ and designated as non-typeable (NT). NT were included in the group of non-PCV13 serotypes.

The original studies^15,16^ were performed in accordance with relevant legislative guidelines and regulations, registered and approved by the Health Care Center of Oeiras that reports to Administração Regional de Saúde (ARS, “Regional Health Administration”) of Lisboa e Vale do Tejo from the Ministry of Health; sampling in 2015–2016 was also registered and approved by the Ethics Research Committee of the NOVA Medical School/Faculdade de Ciências Médicas – Universidade Nova de Lisboa (CEFCM) (47/2014/CEFCM); signed informed consent was obtained from children legal guardians; samples and questionnaires were processed anonymously.

### Statistical analyses

The Welch two-sample t-test was used to test for differences between the child’s mean age, by calendar year, and the child's mean age for the overall period, 1996–2016. Multiple comparisons were controlled for the false discovery rate using the Benjamini–Hochberg method. Differences between the ratio of males to females by calendar year, and differences between prevalence of carriage by year compared to the overall prevalence of carriage, were evaluated using the Pearson's chi-squared test. PCV7 and PCV13 uptake rates were estimated, by year, as the ratio between age-appropriately vaccinated children and the total number of children for which a nasopharyngeal swab was obtained in that year. To investigate whether a temporal trend in PCV7 and PCV13 uptake rates existed, a chi-squared test for trend in proportions was done. A P < 0.05 was considered significant. Statistical analyses were computed in R version 3.6.2 (R Core Team (2020). R: A language and environment for statistical computing. R Foundation for Statistical Computing, Vienna, Austria. URL https://www.R-project.org/).

### Serotype diversity

Diversity (*D*), by calendar year, was estimated using the first three Hills numbers given by Eq. (1);

1 $$^{{\text{q}}} D = \left( {\sum\nolimits_{{i = 1}}^{{\text{R}}} {{\text{p}}_{{\text{i}}}^{{\text{q}}} } } \right)^{{1{\text{ /}}(1 - {\text{q}})}}$$

$\text{p}$ is the proportion of serotypes and $\text{R}$ is the total number of serotypes. The first three Hill numbers—^*0*^*D*, ^1^*D*, and ^*2*^*D*—are related to common diversity indexes: richness, exponential of the Shannon index, and the inverse of the Simpson index, respectively^46^. Note that as $q$ approaches 1, the mathematical limit of Eq. (1) is given by Eq. (2):

2 $${}^{1}D=\text{exp }\left(-{\sum }_{i=1}^{R}\text{log}({p}_{i}){p}_{i}\right)$$

The Hill diversity evenness was estimated using the Eq. (3)^47,48^;

3 $${\text{Hill}}\, {\text{eveness }}=^{{\text{1}}} {\text{D}}\ {{/}}^{{\text{0}}} {\text{D}}$$

The Hill numbers were estimated by calendar year using the observed dataset of pneumococcal serotypes.

Since the total sample size differed by calendar year, we established a common sample size by rarefaction of serotypes richness to normalize the sampling effort. For each year, 1,000 bootstrap re-samples, of equal size, with replacement were obtained. A Hill number was calculated for each re-sample giving a distribution of 1000 Hill numbers for each order.

To test for differences in diversity we calculated the 95% bootstrap confidence interval for the distribution of the differences between the values of each Hill number between the pre- and post-vaccine periods. If the confidence interval did not contain zero the difference was considered significant. A loess smooth curve was fitted to the median diversity values.

To explore the relationship between uptake rates (of PCV7 and PCV13) and diversity, the intrinsic dynamics of the pneumococcal (serotypes) community was taken into account.

A generalized additive model (GAM) was used to study the dependence of diversity, ^*2*^*D*, on three components: PCV7 and PCV13 uptake rates, and the year^49,50^. GAM was chosen given that, by using nonparametric smooth functions of the explanatory variables, it is suitable to deal with nonlinear, non-monotonic relationships between a set of explanatory variables (PCV7 and PCV13 uptake rates and the year), and a response variable (^*2*^*D*). These smooth functions represent an assembly of polynomials joined together by knots. The impact of each of the explanatory variables in the response can be determined by inspecting the partially dependent plots. GAMs were fitted using the R mgcv package.

To investigate which of the distributions, lognormal or Gaussian, provided the best fit to the data, the distribution of ^*2*^*D* and the diagnostic residual plots from the GAM modeling were inspected. To attain a compromise between flexibility and over-fit, the number of knots was limited to 4–5 for all variables. Residuals were inspected to evaluate if the degree of smoothing was appropriate. The smooth term for the *year* used a knot-based penalized cyclic cubic regression spline. This was necessary to guarantee that the predicted values of ^*2*^*D* were positive. Concurvity, to describe nonlinear dependencies among the predictor variables, was inspected (a value of 0 means no concurvity; as it approaches 1, the more obvious concurvity is). The model was fit to ^*2*^*D* from 2001 onwards. The fit was used to predict the values of ^*2*^*D* for the overall period. The predicted values for 2004–2005, 2008 and 2013–2014 (years in which no samples were obtained) were estimated by linear interpolation of the uptake rates.

### Serotype succession and community structure

Serotype abundance distributions (SAD), by year, were fitted to the rank-abundance curves (RACs). The RACs, order the serotypes by their relative abundance from the most to the least abundant serotype. A 95% confidence interval was estimated from the pre-vaccine data after a 1,000 bootstrap re-sampling and by using the bootstrap percentile method. Package Vegan implemented in R was used to fit each SAD. The better fit was given by the distribution that gave the minimum value for the Akaike Informative Criterium (AIC) and the Bayesian Informative Criterium (BIC).

### Similarity and contribution of serotype replacement

To compare pneumococcal serotype communities between calendar years we used the abundance-based Bray–Curtis similarity index (BC) which partitions similarity into two components: (i) balanced variation in abundance, corresponding to serotype replacement, whereby children carrying a serotype at one time point become carriers of a different serotype at the following time point; and (ii) an abundance gradient, corresponding to serotypes carried by children at a given time point ^51^.

We assessed if the similarity between serotypes community decreased with time since PCV7 (considering 2001 as time 0) and PCV13 introduction (considering 2010 as time 0), and whether this decrease could be fitted by an exponential decay model (Eq. 4) which is used in macro-ecology to study, for example, how communities’ similarity changes with distance.

4 $${\text{Similarity}(\text{t})=\text{a exp}}^{-\text{b t}}$$

Similarity was estimated for each year using 1,000 bootstrap re-samples, of equal size, with replacement of the observed dataset. The half-time for replacement was estimated from the fit of the model.

### Composition of pneumococcal serotype communities

To evaluate changes in serotypes composition and abundance over time, hierarchical cluster analysis with average linkage using the Bray–Curtis similarity matrix was carried out. The Bray–Curtis similarity matrix was computed on five replicates of size 275 for each surveyed year. Moreover, multiscale bootstrap of 500 re-sampling was used to compute P for all clusters. The distribution of serotypes that were more relevant for the clustering was compared using a Kruskal–Wallis test. To compute the P for each hierarchical cluster, the package pvclust implemented in R was used.

## Supplementary Information

Supplementary Information.

## Data Availability

The datasets analyzed in this study are available in two previous published papers from our laboratory^15,16^. The data used to support the results of this study can be obtained from the corresponding author upon reasonable request.
