# Supplementary material for: Evidence for the intermediate disturbance hypothesis and exponential decay in replacement in Streptococcus pneumoniae following use of conjugate vaccines
Source: Sci Rep. 2022 May 7;12:7510. doi: 10.1038/s41598-022-11279-5 (PMC9079081; doi:10.1038/s41598-022-11279-5)
Supplement: Supplementary file 1 — Supplementary Information. [file 41598_2022_11279_MOESM1_ESM.pdf]

Evidence for the intermediate disturbance hypothesis and exponential decay in replacement in *Streptococcus pneumoniae* following use of conjugate vaccines

A. Cristina Paulo[1*] and Raquel Sá-Leão[1*]

[1]Laboratory of Molecular Microbiology of Human Pathogens, Instituto de Tecnologia Química e Biológica António Xavier, Universidade Nova de Lisboa Oeiras, Portugal.

Supplementary Table 1. Summary of characteristics of population studied

Characteristic[a]	Study year (sample size)														
	1996	1997	1998	1999	2001	2002	2003	2006	2007	2009	2010	2011	2012	2015	2016
**Sample size**	584	745	780	937	715	717	620	515	470	595	470	434	275	304	311
**Mean age, years ± SD**	3.6±1.6	3.5±1.5	3.5±1.6	3.3±1.6	3.5±1.5	3.5±1.5	3.5±1.5	3.5±1.5	3.5±1.6	3.3±1.6	3.2±1.5	3.3±1.4	3.0±1.5	2.9±1.4	2.8±1.6
**Males, %**	48.6	50.1	47.0	48.9	50.8	55.2	49.7	51.4	51.7	53.8	49.9	50.3	47.9	52.3	53.7
**Carriage, %**	46.9	46.8	58.1	62.8	64.9	66.7	72.7	69.9	62.1	60.7	63.8	64.5	60.4	61.5	61.4
**Participants vaccinated with PCV7, %**	0.0	0.0	0.0	0.0	0.0	11.6	23.2	63.3	71.3	78.3	74.9	59.4	32.7	0.0	0.0
**Participants vaccinated with PCV7 and PCV13, %**	0.0	0.0	0.0	0.0	0.0	0.0	0.0	0.0	0.0	0.0	0.0	13.4	21.8	6.2	0.4
**Participants vaccinated with PCV13, %**	0.0	0.0	0.0	0.0	0.0	0.0	0.0	0.0	0.0	0.0	0.0	5.8	19.7	73.7	84.2
**N. serotypes**	31	30	35	38	31	42	35	33	28	29	27	26	26	24	27

[a]Characteristics of these populations were described in detail[15,16]. PCV7 was commercially available from 2001 to 2010. PCV13 was commercially available from mid-2010 to mid-2015 when it was introduced in the National Immunization Plan.

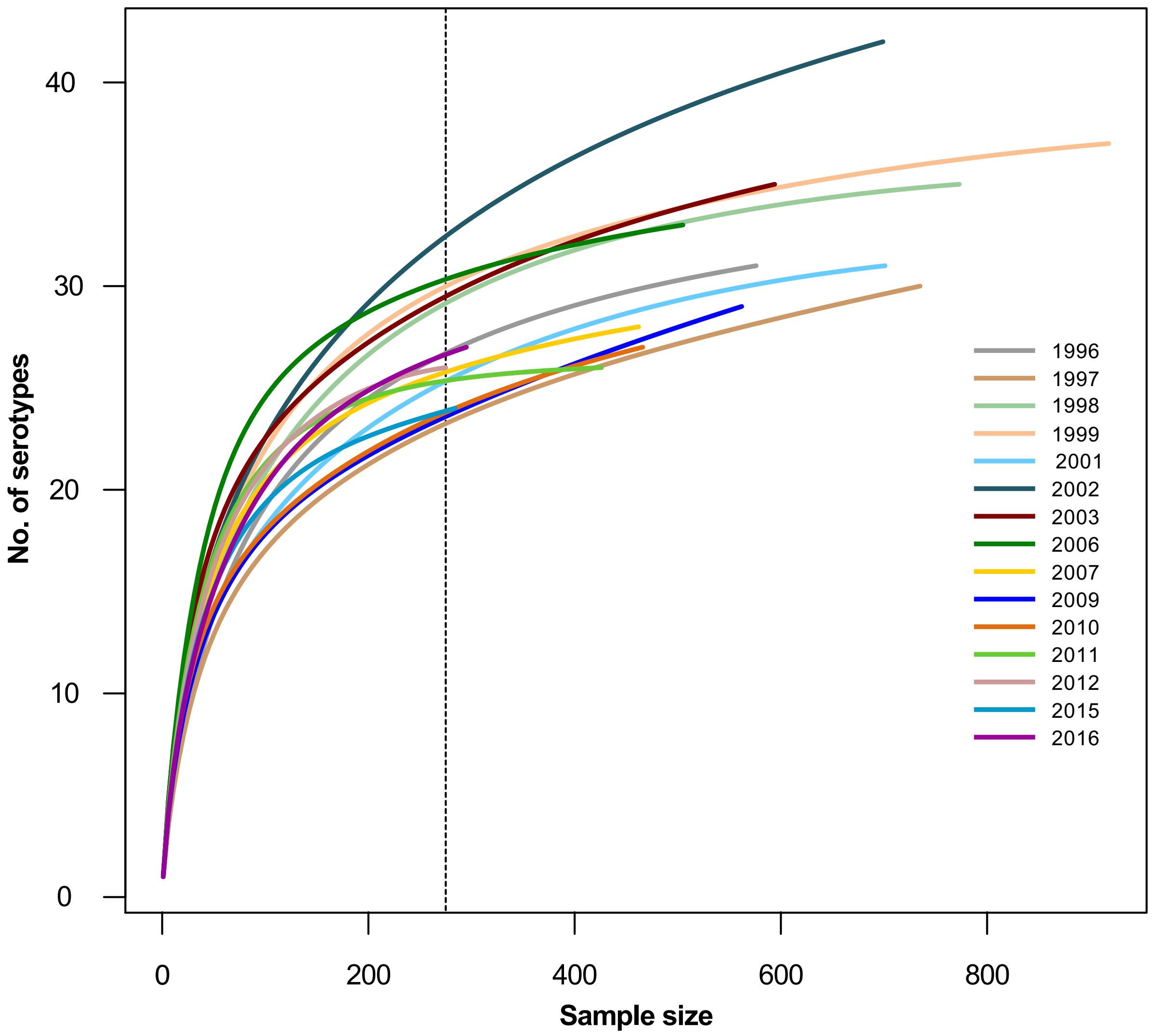

No. of serotypes	Year														
	**1996**	**1997**	**1998**	**1999**	**2001**	**2002**	**2003**	**2006**	**2007**	**2009**	**2010**	**2011**	**2012**	**2015**	**2016**
**Observed**	31	30	35	38	31	42	35	33	28	29	27	26	26	24	27
**Rarefied**	27.6	24.1	30.0	31.1	26.2	33.2	30.2	31.2	26.7	24.2	24.7	26.2	26.0	24.6	27.4

Supplementary Figure 1. Rarefaction of serotypes richness.
Yearly rarefaction curves were estimated. The vertical line indicates the cut-off used to normalize for serotypes' richeness. This cut-off was imposed at the sample size of 275, the minimum sample size which occurred in 2012. At this sample size the estimated number of serotypes for each year can be read in the y-axis. Rarefaction curves were calculated using the package vegan from R[37].

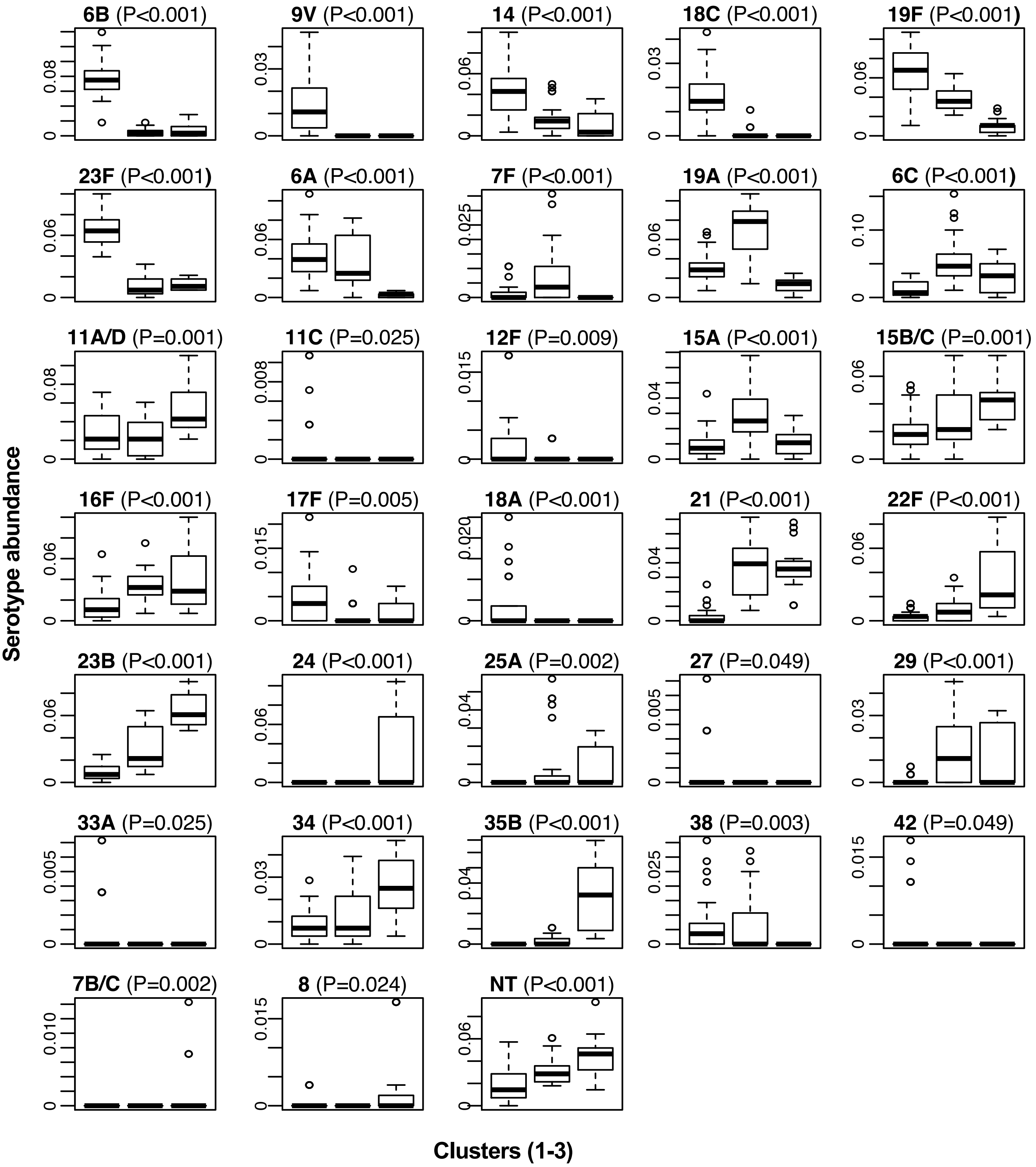

Supplementary Figure 2. Serotype specific abundance per cluster.

To investigate changes in profiles of serotype community composition, hierarchical clustering of the Bray-Curtis similarity indexes was done. Three clusters of pneumococcal serotypes were identified (detailed in Figure 5). Boxplots for each of the serotypes for which a significant difference between the three clusters was detected is shown. In parenthesis the P-value resulting from comparing the three clusters using a Kruskal-Wallis test is shown.
